# Principles for establishment of the stem cell bank and its applications on management of sports injuries

**DOI:** 10.1186/s13287-021-02360-3

**Published:** 2021-05-29

**Authors:** Bao-Shi Fan, Yang Liu, Ji-Ying Zhang, You-Rong Chen, Meng Yang, Jia-Kuo Yu

**Affiliations:** 1 Sports Medicine Department of the Institution of Peking University Third Hospital, Beijing Key Laboratory of Sports Injuries, No. 49 North Garden Road, Beijing, 100191 China; 2 Institute of Sports Medicine of Peking University, No. 49 North Garden Road, Beijing, 100191 China; 3 School of Clinical Medicine, Weifang Medical University, No.7166 West, Baotong Road, Weifang, 261053 Shandong China

**Keywords:** Stem cell, Stem cell bank, Basic principle, Tissue-engineered cartilage, Tissue-engineered meniscus, Tissue-engineered ligament

## Abstract

**Background:**

The stem cells of the stem cell banks have prominent problems for insufficient sources, easy contamination, unstable biological characteristics after serial subcultivations, and high cost.

**Methods:**

After collecting the construction processes of the existing stem cell banks and suggestions from authoritative experts in the past 10 years, 230 reference principles were obtained, and finally, the principles of “5C” for the establishment of modern standardized stem cell banks were summarized, and their related applications on the management of sports injuries were reviewed as well.

**Results:**

The basic principles of “5C” for the establishment of modern standardized stem cell banks include (1) principle of informed consent, (2) confidentiality principle, (3) conformity principle, (4) contamination-free principle, and (5) commonweal principle. The applications of stem cells on repairs, reconstructions, and regenerations of sports injuries were also reviewed, especially in tissue-engineered cartilage, tissue-engineered meniscus, and tissue-engineered ligament.

**Conclusions:**

The proposal of the basic principles of “5C” is conducive to relevant stem cell researchers and clinical medical experts to build modern stem cell banks in a more standardized and efficient manner while avoiding some major mistakes or problems that may occur in the future. On this basis, stem cells from stem cell banks would be increasingly used in the management of sports injuries. More importantly, these days, getting stem cell samples are difficult in a short time, and such banks with proper legal consent may help the scientific community.

## Introduction

Stem cells are a group of primitive cells with the potentials of self-renewal and multidirectional differentiation, and they are also known as “universal cells” and “seed cells” [1, 2]. A stem cell bank is a platform for the large-scale collection, preparation, storage, provision, and research of stem cells, so it is also known as a “life bank” [3].

Since it is difficult to get most stem cells, the standardized production and preservation of stem cells through stem cell banks have become a mainstream trend [4]. The first international stem cell bank was established by the British Government in 2004. Since then, many stem cell banks have been established by governments or research institutions, such as the British Stem Cell Bank, the Korean NIH Stem Cell Bank [5], the Wicell Stem Cell Bank in the University of Wisconsin, and the RIKEN BioResource Center at RIKEN [6, 7] which are mainly for the storage and transfer of stem cell resources.

Although the researches and establishments of stem cell banks in China are late in getting started, the development momentum is rapid. Among them, the National Stem Cell Resource Center established the first clinical-level stem cell bank and clinical-level embryonic stem cell line of China in 2007, and the establishment has actively promoted the sharing of stem cell resources. In 2017, it provided over 200 batches of stem cells for more than 20 research institutions and hospitals for pre-clinical researches or basic researches, actively promoting the translation of stem cells from laboratory to clinic. At present, this center has established nearly 300 clinical-grade human embryonic stem cell lines with definite HLA matching types and nearly 2000 cell lines with different developmental potentials, laying a foundation for standardized and large-scale obtaining of functional cells for clinical application and the development of stem cell drugs. Established in 2000, Peking University Stem Cell Research Center has become a prospective research institute for basic and clinical researches and a training center for stem cell biotechnology in China. Established in 2015, Shanghai East Hospital Biobank is the first standardized biobank, integrating stem cell resources and other sample resources in China. Founded in April 2010, Boyalife is the first and the only clinical-grade stem cell bank in China that has passed the AABB Standard of the United States, the NRL Standard of the World Health Organization, and the CAP Standard of the American Society of Pathology (Table 1). Due to the complexity of building stem cell banks that requires the collaboration of ethics, sociology, medicine, bioinformatics, and many other fields, the domestic and foreign stem cell banks have not yet formed a unified principle or guideline for establishment and application [8].

**Table 1:** The famous stem cell bank established in the world

Name of bank	Stem cell type	Country	Website and/or reference
UK Stem Cell Bank	Comprehensive	UK	http://www.nibsc.org
National Centre for Cell Science Cell Repository	Comprehensive	USA	http://www.nccs.res.in
WiCell International Stem Cell Bank	Comprehensive	USA	https://www.wicell.org/home/stem-cells/stem-cells.cmsx
Cord Blood Registry	Cord blood/cord tissue stem cells	USA	https://parentsguidecordblood.org/cn/banks/cord-blood-registry
Australian Cord Blood Bank	Cord blood	Australia	http://www.stemlife.com.au
BioEden	Child milk tooth stem cells	USA	https://us.bioeden.com
Human Pluripotent Stem Cell Registry	Human pluripotent stem cell	Global registry	http://hpscreg.eu
Swiss Stem Cell Bank	Cord blood	Switzerland	http://parentsguidecordblood.org/en/bank/swiss-stem-cell-bank
China National GeneBank	Comprehensive	China	https://en.genomics.cn/en-gene.html
National Stem Cell Resource Center	Comprehensive	China	http://www.bjscb.cn/dms/gygxb/4.jhtml
Shanghai East Hospital Biobank	Comprehensive	China	http://easthospital.cn/biobank/Home/Index
China Stem Cell Group Boyalife	Cord blood Cord blood	China China	http://www.chinacordblood.org https://www.boyastem.com/about.html

Although stem cells can differentiate into different tissues and cells under certain induced microenvironments, they also tend to age under unstable storage conditions [9]. Aging has a serious impact on all cells and tissues and can cause damages to the stem cell microenvironments [10, 11].

With the increasing cases of sports injury worldwide, issues for the treatments of articular cartilage injuries and the reconstruction of the structure and function of the torn meniscus and ligament need to be put on the agenda to promote recovery of motor function [12]. In the past 20 years, great achievements have been made in the basic and clinical researches of stem cells and tissue engineering procedures in sports medicine. The most worthy of mention is that the rapid development and application of tissue-engineered cartilage (TEC), tissue-engineered meniscus (TEM), and tissue-engineered ligament (TEL) have greatly expanded the feasibility of the clinical application of stem cells in the management of sports injuries [13].

To sum up, in order to meet the needs of clinical treatment and the application markets of stem cells, it is urgent to propose the basic principles of establishing modern standardized stem cell banks. The establishment of stem cell banks and their applications on the management of sports injuries will promote the revolutionary change of sports injury management.

## The principles of “5C” for the establishment of the stem cell bank

**Fig. 1:**
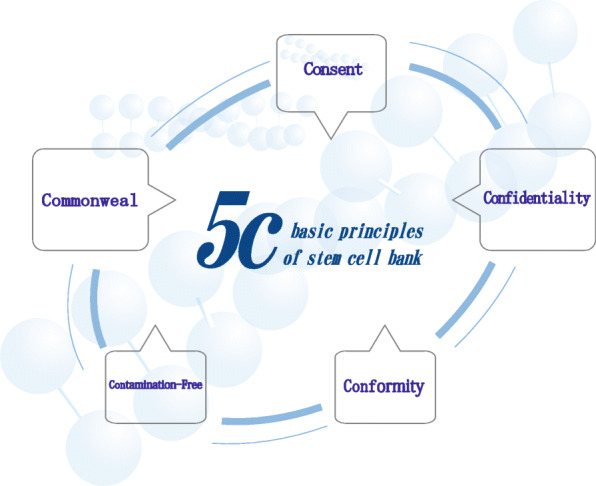
Basic principles of “5C” for the establishment of a modern stem cell bank

The establishment of the stem cell bank is conducive to the researches of stem cells, providing a stable source of seed cells and accelerating the study and development of stem cell technology, which promotes the clinical research processes of stem cells and the conversion applications of stem cells. At the same time, high-quality stem cell banks are not only the premise of clinical applications of stem cell products, but also are the key success factors of the stem cell industry, providing stem cell researches and extensive applications with necessary resources [14]. Therefore, based on a good international environment, speeding up the establishment of the standardized stem cell bank is the core and key to standing at the strategic highland of international high-quality resources. Based on summarizing the relevant international norms and existing experiences in establishing stem cell banks, we proposed the principles of “5C” for the establishment of modern stem cell banks (Fig. 1).

### Principle of informed consent

The principle of informed consent refers to the process, in which patients or healthy volunteers voluntarily donate body tissues or cells and allow researchers to conduct relevant studies [15]. According to the “Informed Consent/Institutional Review Boards: 21 CFR Parts 50/56” issued by the Food and Drug Administration (FDA), donors must first sign an informed consent form. The General Medical Council (GMC) has also stressed the need to ensure that patients have agreed to participate in teaching or researching before any specific test or research. While the types of stem cells and their purposes vary, stem cell banks must ensure that donors are voluntary, documented, and that the information about donors is kept private.

### Confidentiality principle

Confidentiality means that managers of the stem cell bank are forbidden to disclose any specific information about the donors. The sources of the tissues must be kept secretly by the staff of the stem cell bank, to decouple irreversibly the identity of the tissues from the samples. Researchers sometimes hold indirect links (such as ID numbers) between the tissues and the donors that can be used to locate the tissue donors in an emergency [3]. Besides, a sound management system and punitive measures should be made to maximize the prevention of the insiders of the stem cell banks from maliciously disclosing the donors’ information.

### Conformity principle

The conformity of stem cells provides the most basic guarantee for the application of stem cells in basic researches and clinical and commercial applications [16]. To ensure that the quality of stem cells in the bank meets the application standards, the International Stem Cell Bank Initiative (ISCBI) was held in Boston in 2017, and the experts attending the meeting emphasized that the quality control of the stem cell products should be put into the top priority [17]. Therefore, there must be a strict management system to manage and evaluate the quality of the stem cell bank. This system should include the processes of selecting the stem cell donors, storage ethical processes of certain cells, personnel training, the quality and functions of the equipment of the stem cell bank, the culturing and freezing materials used on the stem cells, international regulatory standards, and the guidelines and principles accepted by the stem cell bank industry, such as ISCBI Guidelines and the Organization for Economic Co-operation and Development (OECD) Principles [18, 19].

### Contamination-free principle

In the processes of long-term storage and application of stem cells, it is very important to prevent cross-contamination of cell lines and microbial contamination [20]. First, raw materials should be prevented from exogenous contamination, strict screening standards and procedures should be established for the tissues and cells of donors, and a comprehensive risk assessment for pathogen types (such as HIV) that may introduce contamination risks should be conducted [21, 22]. Secondly, mycoplasma infection should be prevented. The infection rate of mycoplasma reported by cell line testing companies accounts for about 8% of the total test samples [23]. The researchers suggested that the following measures could be taken to prevent the potential contamination risks during the long-term storage of stem cells: (1) select gas-phase liquid nitrogen containers, (2) set the safety helmet when the sample must be immersed in liquid nitrogen containers, (3) use internal thread construction and double bags to store the stem cell samples, (4) ensure the complete closure of the storage containers and the shelf lives of the products, (5) set isolation storage areas to control the spread of pathogens, (6) keep storage containers in a clean environment to reduce the risks of surrounding pollution, (7) ensure clean liquid nitrogen sources, (8) strengthen professional training for laboratory personnel and raise their awareness of preventing microbial pollution, and (9) inspect samples randomly at regular intervals to ensure the purity of the stem cell samples [24].

### Commonweal principle

From 2017 to 2022, the global stem cell market has grown at an annual rate of 36.52%, and the stem cell industry is processing into a period of comprehensive and rapid development. Many experts believe that profiting from human tissues violates the principles of altruistic donation on which we depend and that the economic rewards for donors should not be offered, as this could undermine the principles of informed consent discussed here. Many countries in the world have also made corresponding policies. The European Union required the Stem BANCC to provide specific data on its stem cell production to European researchers at no cost [25]. In the UK, both the GMC and the Medical Research Council (MRC) explicitly prohibited the use of human tissues for economic interests.

## Stem cell drugs

Researches on stem cell drugs not only provided people with rich imagination space, broad application prospects, and important theoretical values, but also contain huge business opportunities. In the USA, federal and non-federal funding agencies have spent billions of dollars on clinical trials, and they believe that stem cell drugs are a promising therapy for Alzheimer’s and Parkinson’s disease-related disorders [22]. Therefore, stem cell drugs have attracted the attention and investment of businesses and governments.

China has also laid down a series of national policies to promote the clinical application of stem cells. In 2017, China formulated the technical management specifications for the transplantation of hematopoietic stem cells. On August 21, 2015, the National Health Commission of the People’s Republic of China and the National Medical Products Administration (NMPA) jointly made the policy for the administration of clinical researches or clinical trials on stem cell therapies. By March 19, 2020, new five clinical research institutions and four clinical research projects on stem cells had been registered in China. Table 2 shows that many kinds of stem cell drugs had been approved in the world till now. As of 2016, about 1000 clinical trials on stem cells had been conducted globally [26]. And excitedly, some stem cell-based products have been approved in some countries (Table 2).

**Table 2:** Internationally approved stem cell drugs

Year	Corporation	Stem cell type	Clinical application	Name of bank
2009.12	Prochymal/Osiris Therapeutics, Inc., USA	Mesenchymal stem cells from human allogeneic bone marrow	Graft versus host disease (GVHD) and Crohn’s disease	American Food and Drug Administration (FDA)
2010.07	MPC/Mesoblast	Autologous mesenchymal precursor cells	Bone reconstruction	Production permit of Therapeutic Goods Administration (TGA) in Australia
2011.07	Hearticellgram-AMI/FCB-Pharmicell	Autologous bone marrow mesenchymal stem cells	Acute myocardial infarction	Food and Drug Administration in South Korea
2011.11	Hemacord/New York Blood Center	Cord blood hematopoietic progenitor cells	Hereditary or acquired hematopoietic disease	Biological License of American Food and Drug Administration (FDA)
2011.11	MultiStem/Athersys Inc., USA	Allogeneic bone marrow stem cell	Type I mucopolysaccharidosis	American Food and Drug Administration (FDA)
2012.01	Cartistem/Medi-post	Mesenchymal stem cells from the cord blood	Degenerative arthritis and cartilage damage in the knee joint	Food and Drug Administration in South Korea
2012.01	Cuepistem/Anterogen	Mesenchymal stem cells from autologous fat	Crohn’s disease complicating anal fistula	Food and Drug Administration in South Korea

## Multiple applications of stem cell banks in the management of refractory sports injuries

The stem cell therapy products represented by mesenchymal stem cell (MSC) preparation are expected to become the third type of disease treatment products after drugs and medical devices and may play a good effect in some diseases that cannot be cured by existing treatment methods, opening up new ways for diseases [27]. In the field of sports medicine, the combination of stem cells and tissue engineering scaffolds provides a new therapeutic option for the regeneration and repair of the cartilage, meniscus, and ligament.

### Tissue-engineered cartilage

Articular cartilage plays an important role in joints. But due to the lack of nerves and blood vessels, articular cartilage regeneration is very limited [28]. The development and progress of tissue engineering have brought new hope for the repair of cartilage injury and even myocardial infarction [29]. And the first step in tissue-engineered cartilage research is to identify appropriate seed cells to promote cartilage regeneration. For cartilage regeneration, the most used therapeutic stem cells are MSCs [30–36].

### Tissue-engineered meniscus

Stem cells are popular in the field of tissue-engineered meniscus due to their autocrine ability of cytokine and growth factors that favors tissue regeneration [37]. In recent years, stem cell-based TEM approaches have been widely used in meniscus regeneration, which obtained encouraging results of meniscus regeneration. Chang et al. have demonstrated that protein-releasing polymeric scaffolds inducing endogenous stem cells regenerated the knee meniscus, with the maintenance of the meniscal structure and function, providing evidence for a chondroprotective effect in a sheep model. Of note, this highlights the key roles of MSCs and growth factors in successful meniscal healing to generate normal cellular phenotypes [38].

TEM also brought good news to patients in clinics. Whitehouse et al. tested MSCs/collagen scaffold in a sheep meniscal tear model with promising results after 13 avascular meniscal tears and reported a case of 5 patients where undifferentiated mesenchymal stem cells were injected onto a collagen scaffold and sutured into an avascular meniscal tear. Three out of five patients reported positive outcomes beyond 12 months with significantly improved clinical scores and subsequent magnetic resonance imaging (MRI) scans. We knew that undifferentiated autologous MSCs seeded onto collagen scaffolds can be safely implanted into patients with a torn meniscus, so avoiding the need for meniscectomy [39]. With the advancements in tissue engineering and stem cell-based technologies, new therapeutic options for patients will potentially emerge.

### Tissue-engineered ligament

Stem cells are seed cells emerging from tissue-engineered ligaments in recent years. Compared with adult cells, stem cells have many advantages. Taking mesenchymal stem cells as an example, they have the potential of multi-differentiation [40] and have been widely used in animal models of bone injury, ligament injury, and other diseases [41, 42]. Studies have found that bone marrow mesenchymal stem cells can be directed to differentiate into ligamentoid cells, promote vascularization of ligament tissue, and repair and regenerate damaged ligaments [43].

In summary, stem cells represent a significant cell source for tissue engineering under certain conditions, including the presence of suitable microenvironments and bioactive factors. However, stem cells still have defects such as induced differentiation, expensive proliferation, and low efficiency in vitro, so further research is needed.

## Prospects of the application of stem cell banks in the management of refractory sports injuries

Although currently there are not many applications from stem cells clinically available for sports injury treatment, the rapid development and application of TEC, TEM, and TEL have greatly expanded the feasibility and innovation of the clinical application of stem cells in the management of sports injuries. The researches on stem cell are undoubted of significance for the management of athletes’ refractory injuries and have potential therapeutic values for injuries of the cartilages, menisci, and ligaments. At the same time, with the continuous development such as 3D printing, the future stem cell biotechnology relying on the stem cell bank may completely overturn the traditional medical concepts and bring unexpected “surprises” and “rewards” to the treatment of refractory sports injuries. Therefore, stem cells have the potential to change the fate of each of us and even the entire human society.

But there is a long way to go, such as how to overcome immune rejection and control the optimal growth, proliferation, and differentiation conditions of stem cells in vivo. The modernization and systematic management of the stem cell banks have maximized the application boundary of stem cells and brought infinite benefits to human beings, but at the same time, there are some hidden dangers. Along with the establishment of stem cell banks, there will inevitably be some worrying things. If people use stem cell technology to produce stem cell stimulants, they will transplant the stem cells of mammals with sports specialties to athletes to improve their competitive performances. This will involve a series of ethical issues that even go against the Olympic spirit of “fair competition.” In the future, it will be an important research direction to strengthen the ethical review of the applications of stem cell banks and improve the detection technology.

## Data Availability

Not applicable.

## Abbreviations

ACL: Anterior cruciate ligament
BMMSCs: Bone marrow-derived MSCs
FDA: Food and Drug Administration
GMC: General Medical Council
HLA: Human leukocyte antigen
ISCBI: International Stem Cell Bank Initiative
MRC: Medical Research Council
MRI: Magnetic resonance imaging
MSCs: Mesenchymal stem cells
OECD: Organization for Economic Co-operation and Development
PBMSCs: Peripheral blood-derived MSCs
TEC: Tissue-engineered cartilage
TEL: Tissue-engineered ligament
TEM: Tissue-engineered meniscus
